# The New Paradigms in Clinical Research: From Early Access Programs to the Novel Therapeutic Approaches for Unmet Medical Needs

**DOI:** 10.3389/fphar.2019.00111

**Published:** 2019-02-13

**Authors:** Cristina Scavone, Gabriella di Mauro, Annamaria Mascolo, Liberato Berrino, Francesco Rossi, Annalisa Capuano

**Affiliations:** Campania Regional Centre for Pharmacovigilance and Pharmacoepidemiology, Department of Experimental Medicine, Section of Pharmacology “L. Donatelli”, University of Campania “Luigi Vanvitelli”, Naples, Italy

**Keywords:** clinical research, early access programs, novel therapeutic approaches, unmet medical needs, challenges

## Abstract

Despite several innovative medicines gaining worldwide approval in recent years, there are still therapeutic areas for which unsatisfied therapeutic needs persist. For example, high unmet clinical need was observed in patients diagnosed with type 2 diabetes mellitus and hemophilia, as well as in specific age groups, such as the pediatric population. Given the urgent need to improve the therapy of clinical conditions for which unmet clinical need is established, clinical testing, and approval of new medicines are increasingly being carried out through accelerated authorization procedures. Starting from 1992, the Food and Drug Administration and the European Medicines Agency have supported the so-called Early Access Programs (EAPs). Such procedures, which can be based on incomplete clinical data, allow an accelerated marketing authorization for innovative medicines. The growth in pharmaceutical research has also resulted in the development of novel therapeutic approaches, such as biotech drugs and advanced therapy medicinal products, including new monoclonal antibodies for the treatment of asthma, antisense oligonucleotides for the treatment of Duchenne muscular dystrophy and spinal muscular atrophy, and new anticancer drugs that act on genetic biomarkers rather than any specific type of cancer. Even though EAPs and novel therapeutic approaches have brought huge benefits for public health, their implementation is limited by several challenges, including the high risk of safety-related label changes for medicines authorized through the accelerated procedure, the high costs, and the reimbursement and access concerns. In this context, regulatory agencies should provide the best conditions for the implementation of the described new tools.

## Introduction

The development of a new medicine is a long, expensive and risky process. The entire time that passes from the R&D phase until the drug’s marketing approval can last up to 15 years, and it is characterized by extremely high costs, usually exceeding $1.2 billion (Saadi and White, 2014). At the initial phase, before clinical trials can be carried out in humans, preclinical studies on animals, which are mainly aimed to characterize the mechanisms of action, the toxicity, the dosage or route of administration of the new medicine, are provided (Andrade et al., 2016). Based on the positive results of preclinical research, the new drug can be evaluated in humans during the four main phases of the clinical development. In particular, phase I–III studies are those that evaluate the efficacy and safety profile of the new drug in humans until the marketing authorization. Differently from phase I studies, which involve healthy patients and whose study design is relatively simple, phase II and III studies enroll patients affected by the disease for which the new drug is indicated, and are characterized by a more structured study design, which is usually randomized and controlled (randomized controlled trials, or RCTs). Once the new medicine is authorized, based on data demonstrating the positive benefit/risk profile, the real-world effectiveness and safety of the drug is assessed during phase IV studies (Auricchio et al., 2017; Mascolo et al., 2017). In this last phase, pharmacovigilance is included. Therefore, the “clinical value” of a new drug is observed during a rigorous clinical program, in which it is compared with the best available treatments, if they exist (Morgan et al., 2008). Apart from the traditional design of RCT, in recent years further study designs, including umbrella, basket and platform trials, were developed and applied to new therapies, especially in the area of oncology research (Simon, 2017). The reason for the introduction of these new study designs lies in the discovery of cancer genomic features and consequently in the development of target therapies able to recognize specific oncogenes.

Despite RCTs representing the highest level of the evidence-based medicine pyramid, they suffer from several limitations in predicting effectiveness, which mainly include the limited duration, the highly controlled setting, and the exclusion of frail populations, including children, the elderly, pregnant women, as well as patients affected by multiple diseases and those receiving concomitant medications (Wang et al., 2018). Furthermore, during the premarketing phase, the efficacy and safety data are frequently evaluated using a non-inferiority or equivalence study design and surrogate outcomes. Considering these limitations, the real value of a new drug can be confirmed only when it will be used in real life conditions (Oyinlola et al., 2016).

## Unmet Clinical Needs and Early Access Programs

**FIGURE 1:**
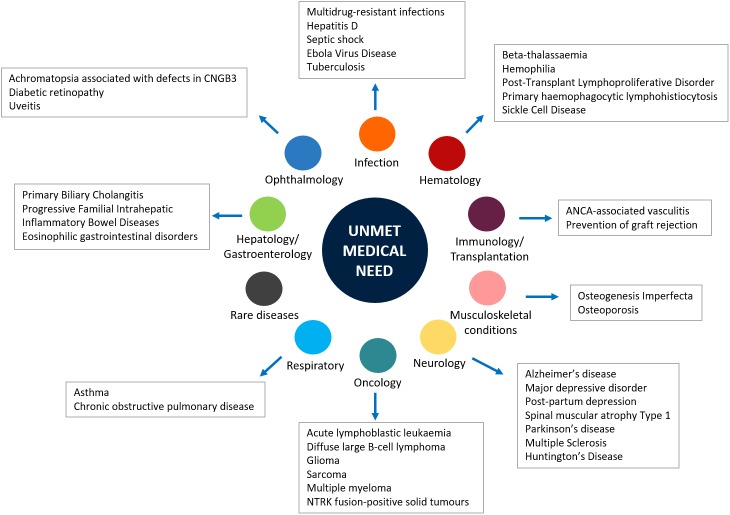
Main unmet medical needs in therapeutic areas.

Generally, the main objective of the development of a new medicine is to respond to an unmet medical need (Kaplan et al., 2013; U.S. Department of Health and Human Services, 2014). Indeed, when a new medicine obtains the marketing authorization, the respective regulatory agency performs a global evaluation of the clinical benefit associated to the new drug as well as an evaluation of the therapeutic need. This latter action is carried out through the analysis of the global burden of the disease for which the new drug is indicated and the prediction of the trend of the disease’s burden. Both evaluations are based on epidemiological and demographic data. Those assessments include the evaluation of the Disability-Adjusted Life Year (DALYs), which is a measure of mortality and disability associated with a clinical condition, and the efficacy and safety profiles of medicines already available for treating that disease. In addition, unmet clinical need is further demonstrated when the new drug has significant effects on serious outcomes, if it demonstrates efficacy in patients who do not tolerate or do not respond to the available drug therapies, and better safety/compliance/drug-drug interactions profiles (Ujeyl et al., 2012). Although several innovative therapies were recently approved worldwide, there are still therapeutic areas for which unsatisfied therapeutic needs persist (Figure 1). Indeed, the so-called “white spots” – clinical conditions for which no efficacious treatments are approved – still exist among pharmaceutical pipelines (Papaluca et al., 2015). For example, a high unmet clinical need was observed in patients diagnosed with type 2 diabetes mellitus (T2DM), which represents an increasing health concern worldwide and is a leading risk factor for cardiovascular diseases. Bennett et al. (2014) evaluated outcomes in T2DM patients in United Kingdom general practice and described the unmet clinical need in T2DM as the failure to meet targets for blood pressure, total cholesterol, or glycated hemoglobin levels. The authors suggested that conventional therapies for T2DM are not able to manage all the clinical aspects of this condition; thus, there is a need for new therapeutic agents in order to alleviate the health and economic burden associated with T2DM (Bennett et al., 2014). Unmet needs were identified also in the management of hemophilia. Indeed, even though several improvements were made in this field, the development of inhibitors, namely the immune response that occurs in patients treated with clotting factor concentrates, negatively affects treatment (Dargaud et al., 2016). A high rate of unmet clinical needs is also found in specific age groups, such as the pediatric population. For these patients, the lack of data from the premarketing development, including the lack of age-specific dose and long-term efficacy and safety data, has significant impact on pharmacological treatments. This is particularly true in the case of psychiatric disorders, which still represent a challenge in children and adolescents (Pozzi et al., 2018; Rafaniello et al., 2018). Indeed, drugs indicated for these conditions are frequently used as off-label in children, based on data from RCTs that have involved only adult patients (Capuano et al., 2014; Persico et al., 2015; Rafaniello et al., 2016). Similarly, the therapeutic area of respiratory diseases, such as asthma, still represents a concern for children. Once again, this is mainly related to the lack of clinical efficacy and safety evidence, but also to the limited availability of non-steroid-based alternative therapies for children < 6 years of age (Lindly et al., 2016).

**FIGURE 2:**
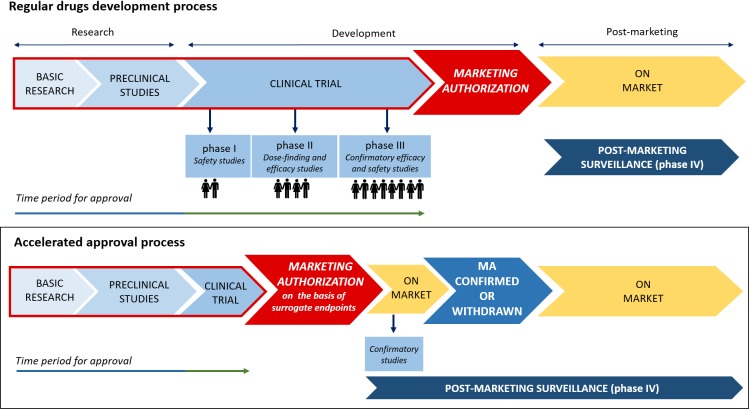
Traditional vs. accelerated development and approval process.

Given the urgent need to improve the therapeutic armamentarium of particular clinical conditions for which unmet clinical need is confirmed, clinical testing and approval of new medicines are increasingly carried out through accelerated authorization procedures. As a matter of fact, both the Food and Drug Administration (FDA) and the European Medicines Agency (EMA) have supported the so-called “Early Access Programs” (EAPs). Since 1992, the FDA has introduced the “Priority Review” or “Fast Track,” designed to make available new drugs for the treatment of serious or life-threatening diseases (conditions associated with morbidity that have significant impact on specific factors, such as survival or day-to-day functioning) without therapeutic alternatives. For these drugs the “breakthrough designation” can be expected. The FDA’s “Fast track” imposes on the pharmaceutical company lower standards than the regular procedure (U.S. Department of Health and Human Services, 2014). Similarly, in the European context, specific regulatory procedures, including approval under exceptional circumstances as well as conditional and accelerated approval, have been introduced in order to accelerate the marketing authorization of a new drug. With such procedures, the marketing authorization application should be based on incomplete clinical data (even data from phase II studies), and its evaluation can be reduced from 210 to 150 days if the applicant provides sufficient justification for an accelerated assessment (Figure 2; European Medicines Agency, 2018). Furthermore, the EMA has recently introduced new tools to support the EAPs, Adaptive Licensing and PRIority Medicines (PRIME). The first one is a prospective authorization process that allows an initial approval based on limited scientific evidence only for a small group of patients. When further evidence is collected, the drug’s access can be expanded to larger patients’ populations (Vella Bonanno et al., 2017). In 2016, EMA implemented the PRIME scheme, which offers early and enhanced dialog between the EMA and the pharmaceutical industry in order to enhance development plans and speed up the evaluation process (European Medicines Agency, 2017). In Table 1, a few examples of medicines evaluated within the PRIME scheme are reported.

**Table 1:** Medicines evaluated in PRIME scheme.

Medicine	Therapeutic indications	Reason for disease’s unmet medical need
Avacopan	AAV	Rare disease
Axicabtagene ciloleucel (CAR-T)	DLBCL	Rare disease
Emapalumab	HLH	Rare disease
Onasemnogene abeparvovec	SMA Type 1 in pediatric patients	Rare disease
Tisagenlecleucel	R/R ALL	Less than 10% of patients achieve 5-year overall survival. Moreover, the R/R ALL is associated with high relapse rates (Papadantonakis and Advani, 2016)
Aducanumab	Alzheimer’s disease	Current drugs improve symptoms, but do not have profound disease-modifying effects (Citron, 2010)
Asunercept	Glioblastoma	Rare disease
Brexanolone (Allopregnanolone, SAGE-547)	PPD	There is a need for new treatment options for mothers suffering from the disorder (Kanes et al., 2017)
Chimeric 2’-O-(2-methoxyethyl) modified oligonucleotide targeted to huntingtin RNA (RO7234292)	Huntington’s Disease	Rare disease
Deoxycytidine / deoxythymidine	TK2	Rare disease
Givosiran	Prevention of acute attacks of hepatic porphyria	Rare disease
Lumasiran	PH1	Rare disease
MV-CHIK vaccine	Prevention of Chikungunya fever	Rare disease
Mycobacterium tuberculosis (MTBVAC)	TB Vaccine	Immune responses of human newborns and infants are distinct and cannot be predicted from those of human adults or animal models (Sanchez-Schmitz and Levy, 2011)
Olipudase alfa	Non-neurological manifestations of acid sphingomyelinase deficiency	Rare disease
Polatuzumab vedotin	R/R DLBCL	Rare disease
Rapastinel	Adjunctive treatment of MDD	Many patients with MDD fail to achieve a complete response with antidepressant medications and experience periods with residual symptom burdens (Rush et al., 2006)
Recombinant Vesicular Stomatitis Virus with Envelope Glycoprotein replaced by Zaire ebolavirus (Kikwit Strain) Glycoprotein	Vaccination against Ebola	Many survivors and their relatives continue to experience stigma and social isolation. Moreover, patients have health, psychological, and social needs (Calnan et al., 2017)
Seladelpar (MBX-8025)	Primary Biliary Cholangitis	Rare disease
Setmelanotide	Obesity and control of the hunger associated with deficiency disorders of the MC4R receptor pathway	Rare Genetic Disorders
Setrusumab	OI types I, III, and IV	Rare disease
Tasadenoturev	Recurrent glioblastoma	Rare disease
Vocimagene amiretrorepvec	Treatment of high-grade glioma	Rare disease
Voxelotor	SCD	Many patients experience poor clinical outcomes in short and longer term (Sarri et al., 2018)


*AAV, active ANCA-associated vasculitis (including granulomatosis with polyangiitis and microscopic polyangiitis); DLBCL, large B-cell lymphoma; HLH, primary hemophagocytic lymphohistiocytosis, SMA: spinal muscular atrophy; R/R ALL, relapsed or refractory B cell acute lymphoblastic leukemia; PPD, Postpartum depression; TK2, Thymidine Kinase 2 Deficiency; PH1, Primary Hyperoxaluria Type 1; TB Vaccine, Active immunization against tuberculosis disease; R/R DLBCL, relapsed and refractory patients with diffuse large B cell lymphoma; MDD, major depressive disorder; OI, Osteogenesis Imperfecta; SCD, Sickle Cell Disease.*

## Novel Therapeutic Approaches

Whilst regulatory agencies have promoted EAPs, which have increased over time during the past 25 years (Beaver et al., 2018), the growth in pharmaceutical research has resulted in the discovery and development of novel therapeutic approaches, mainly represented by biotech drugs and gene-, cell- and tissue-therapies. Starting from the marketing approval of the first monoclonal antibody in 1986, the entire class of biotech drugs has grown significantly. In 2013, the global sales revenue for all mAbs was almost $75 billion, representing half of the total sales of all biopharmaceutical products (Ecker et al., 2015). Most of these novel therapeutic approaches are indicated for the management of different diseases with recognized unmet medical needs. For example, until recently the pharmaceutical armamentarium of asthma included muscarinic antagonists, beta2-agonists and corticosteroids, but approximately half of patients do not adequately respond to the available therapies. For this condition, four monoclonal antibodies (mAbs) recently obtained the marketing authorization (Bateman et al., 2008; Swedin et al., 2017). Omalizumab is a recombinant humanized mAb that binds to the F_C_ portion of the IgE antibody, preventing the binding of IgE to high-affinity IgE receptors on mast cells and basophils and the release of inflammatory mediators induced by allergen exposure (Thomson and Chaudhuri, 2012; Sattler et al., 2017). Mepolizumab, benralizumab, and reslizumab are anti-IL-5 mAbs that bind IL-5Rα, causing apoptosis of eosinophils and basophils through the antibody-dependent cellular cytotoxicity (Farne et al., 2017; Chung, 2018). Currently, these mAbs are indicated as add-on treatment in adults, adolescents and children aged ≥6 years with severe persistent allergic (omalizumab) and refractory eosinophilic (mepolizumab, benralizumab, and reslizumab) asthma inadequately controlled with other available treatments.

Among novel therapeutic approaches, Advanced Therapy Medicinal Products (ATMPs), including cell-, gene-, and tissue-engineered therapies, are particularly noteworthy, especially in the field of neurodegenerative and neuromuscular diseases, for which effective treatments have been limited. The antisense oligonucleotides (ASOs) are synthetic single-stranded strings of nucleic acids, which selectively bind to specific pre-messenger ribonucleic acid (pre-mRNA)/mRNA sequences leading to an alteration of the synthesis of proteins. The FDA approved in 2016 two ASOs for the treatment of Duchenne muscular dystrophy and spinal muscular atrophy. Their role was envisaged also in further clinical conditions, such as Huntington’s disease, amyotrophic lateral sclerosis and Alzheimer’s disease (Wurster and Ludolph, 2018). It should be highlighted that in most cases ATMPs are indicated for the treatment of very rare conditions but, unlike traditional drugs that are administered with a certain frequency, they require administration only once, which is then able to provide for years or for the entire life the biological activity and clinical benefit. This is the case, for example, with axicabtagene ciloleucel, also known as CAR-T therapy, which is indicated for the treatment of diffuse large B-cell lymphoma.

Lastly, a new generation of anticancer drugs is going to be developed. Recently, Drilon et al. (2018) have performed a phase I/II trial, using a basket design, in order to evaluate the safety and efficacy of larotrectinib, a selective inhibitor of TRK proteins, in 55 patients with NTRK genetic alterations regardless of tumor types (Ghosh and Tabrizi, 2017). According to its peculiar pharmacodynamic features, larotrectinib acts on genetic biomarkers rather than any specific type of cancer. On the basis of the efficacy and safety data of this cutting-edge cancer therapy, in November 2018 the FDA granted Priority Review for larotrectinib for the treatment of adult and pediatric patients with locally advanced or metastatic solid tumors harboring an NTRK gene fusion (Drilon et al., 2018). Furthermore, in cancer settings as well as in other therapeutics areas, new drug-drug and drug-antibody combinations are increasingly emerging as therapeutic approaches to fulfill several unmet needs. For example, in the treatment of hemophilia the conjugation of immunogenic peptides to a non-immunogenic protein carrier, such as in the case of the recombinant FVIII–Fc fusion protein, decreases the immunogenicity of FVIII, leading to better clinical outcomes. Similarly, in the treatment of leukemia, the conjugation of PEG to the native asparaginase enzyme leads to fewer allergic reactions than asparaginase enzyme alone (U.S. Food and Drug Administration, 2018). New antibody-drug conjugates (ADCs), mainly indicated for the treatment of hematological and solid malignancies, are currently in the late stage of clinical development but are already showing promising results (Lieuw, 2017). Furthermore, the combination therapy of multiple drugs turned out to be useful also in the treatment of T2DM or hypertension, leading to better glycemic, and blood pressure control. Despite these advantages, it should be stressed that the combination therapy of multiple drugs might increase treatment complexity and adverse events that affect long-term adherence (Mittermayer et al., 2015; Parslow et al., 2016). For these new drug-drug and drug-antibody combinations, a regular approval procedure is required, unless conditions for accelerated approval (confirmation of unmet need) exist. This is currently true also for new combination therapies, such as the polypill indicated for the treatment of hypertension or ADCs used in cancer settings; therefore, even though the individual drugs are already authorized, new approval is required. The novel therapeutic approaches do not necessarily refer solely to new medicines but also to already-existing ones that are used for different indications. This is the case, for example, with exenatide, a glucagon-like peptide-1 receptor agonist used for the treatment of T2DM, whose role is currently being evaluated for the treatment of Parkinson’s disease (PD). Although available medications for PD have strong therapeutic effects, they are not able to stop the progression of the disease. Exenatide has demonstrated a neuro-protective effect in preclinical models of PD. In a clinical study that enrolled 62 patients with moderate PD, conflicting results were obtained (Cimmaruta et al., 2018). New drugs, including those based on pluripotent stem cell therapy, are currently evaluated in clinical programs for the management of PD. Recent literature indicates that stem cells might represent a potential approach for developing novel treatment strategies for PD in humans (Athauda et al., 2017; Yasuhara et al., 2017).

## The Challenges of New Paradigms in Clinical Research

Even though the new tools of clinical research and the discovery of novel therapeutic approaches have brought huge novelties and benefits, their implementation is limited by several challenges. First, EAPs should be applied only in fully justified circumstances in order to ensure the patient’s safety. Furthermore, since the majority of EAPs are based on surrogate endpoints, the use of the new medicine in real life conditions must prove that it is able to improve the patient’s health status and quality of life (patient-relevant endpoint), demonstrating significant benefit and a good safety profile. However, the interpretation of efficacy and safety data from clinical studies, especially when they are not complete, is extremely critical. Indeed, several medicines that underwent fast-track procedures by the FDA were withdrawn for safety reasons; this was the case with valdecoxib and rofecoxib, which were withdrawn due to an increased risk of cardiovascular events, and cerivastatin, which was associated with an increased risk of hepatic adverse events (Zhang et al., 2018). Similarly, the results of a recent study by Mostaghim et al. (2017) revealed that accelerated pathway drugs are associated to higher risks of safety-related label changes, including changes to boxed warnings and contraindications, compared with non-accelerated pathway drugs (Chary, 2016).

Another peculiar aspect of recently authorized therapies is their extremely high costs. As previously reported, except for rare cases, the novel therapeutic approaches are mainly represented by biological products and advanced therapies (Mostaghim et al., 2017). Those substances are characterized by highly complex development procedures as well as by large-scale molecules with huge heterogeneity (Abou-El-Enein et al., 2016; Scavone et al., 2017a,b). Given these intrinsic characteristics and considering the difficulties in their development, those medicines are defined by higher costs compared to traditional ones. For example, the new monoclonal antibodies targeting the PCSK9 had an average cost that exceed 8,000€/patient/year, while statins cost almost 50€/patient/year (Agenzia Italiana Del Farmaco, 2017; Scavone et al., 2017c). Understandably, the investment made in R&D should be at least partially reclaimed. In order to overcome issues related to the high costs of innovative medicines, each EU member state has implemented regulatory tools for reimbursement of medicinal products, the so-called Managed Entry Agreements (MEAs). These contracts, which are stipulated between the pharmaceutical industry and the payers/regulatory agencies, allow conditional access to the market for new drugs with unclear efficacy and safety profiles. Therefore, their objective is to improve access to new medicines in the context of uncertainty and high price. The use of MEAs has been implemented in several countries, including the United Kingdom (where MEAs are defined as “patient access schemes”), Italy, Belgium, and Australia. For example, in Italy the modulation of price and reimbursement schemes are planned by using two categories of MEAs, which include the health outcomes-based agreements (payment by result, risk sharing, and success fee) and the finance-based agreements (cost-sharing agreements and capping) (Mallya et al., 2017; Scavone et al., 2018).

Lastly, apart from the cost and safety concerns of new medicines approved through EAPs, a further challenge is represented by the lack of knowledge and infrastructure necessary for the storage, distribution and administration of the new therapeutics; this is true especially for the advanced therapy medicinal products (cell-, gene-, and tissue-engineered therapies) (Ferrario et al., 2017).

## Conclusion

Despite the progress made in the field of clinical research, unmet therapeutic needs are still identified in several clinical areas (Miller, 2009; Taiwo et al., 2010; Aceves, 2014; Markowitz, 2015; Morrow et al., 2017). It is notable, for instance, that multidrug-resistant infections are rapidly increasing worldwide, but very few antibiotics able to treat these infections are currently under development. Therefore, this research area should be prioritized in the pharmaceutical industries’ pipeline.

Recent developments in clinical research have also placed a series of challenges for regulatory agencies, which are required to create the best conditions for the implementation of the described new tools. Although the application of EAPs improves the patient’s access to new medicines, obtaining new data on their real effectiveness and safety might be a concern. In this context, at least for EU countries, it is expected that the forthcoming application of Regulation No. 536/2014 on clinical trials will facilitate the conduct of clinical trials also in real-life conditions. Indeed, the active participation of patient organizations in clinical research will lead to better study designs, but also to improved reliability of study results and better applicability to patients in the real world. This will lead to increased knowledge of the effectiveness and safety profile of drugs also approved through EAPs. Furthermore, since Regulation No. 536/2014 introduces an authorization procedure based on a single submission via a single EU portal, clinical trial data on effectiveness and safety will be easily accessible. A further step for the better collection of clinical data would be the implementation of a health database and registries, whose potential are undisputed.

Finally, regarding the high costs characterizing new therapeutic approaches, it is advisable that price and reimbursement tools (i.e., MEAs), such as those used in several EU and non-EU countries, may also be applied in other countries. This will lead to an increased access to innovative therapies. Lastly, in order to further implement clinical research, especially in the field of ATMPs, a strengthening in research infrastructures and research training is what is required.

## Author Contributions

CS, GdM, AM, LB, FR, and AC drafted the work and revised it for important intellectual content, made substantial contributions to the acquisition, analysis, or interpretation of data for the work, wrote the manuscript, approved the final version of the manuscript to be published, and agreed to be accountable for all aspects of the work in ensuring that questions related to the accuracy or integrity of any part of the work are appropriately investigated and resolved. FR and AC developed the concept.

## Conflict of Interest Statement

The authors declare that the research was conducted in the absence of any commercial or financial relationships that could be construed as a potential conflict of interest.
